# Gingerol and/or sorafenib attenuates the DAB-induced HCC and hepatic portal vein dilatation via ATG4/CASP3 and COIIV/COX-2/NF-κB expression

**DOI:** 10.1007/s12032-023-02284-3

**Published:** 2024-01-16

**Authors:** Afrah Fatthi Salama, Ali H. El-Far, Esraa Ali Anbar, Sabry Ali El-Naggar, Rami M. Elshazli, Alaa Elmetwalli

**Affiliations:** 1 Biochemistry Section, Chemistry Department, Faculty of Science, Tanta University, Tanta, 31527 Egypt; 2 Department of Biochemistry, Faculty of Veterinary Medicine, Damanhour University, Damanhour, 22511 Egypt; 3 Physiology Department, Faculty of Science, Tanta University, Tanta, 31527 Egypt; 4 Biochemistry and Molecular Genetics Unit, Department of Basic Science, Faculty of Physical Therapy, Hours University, New Damietta, Egypt; 5 Department of Clinical Trial Research Unit and Drug Discovery, Egyptian Liver Research Institute and Hospital (ELRIAH), Mansoura, Egypt; 6 Microbiology Division, Higher Technological Institute of Applied Health Sciences, Egyptian Liver Research Institute and Hospital (ELRIAH), Mansoura, Egypt

**Keywords:** Sorafenib, Gingerol, HCC, DAB, COIIV/COX-2/NF-κB

## Abstract

Ginger (Gin) has numerous therapeutic properties. One of Gin’s most potent components is 6-gingerol, a naturally occurring phenol. This study aimed to investigate the therapeutic impact of gingerol and/or sorafenib on the ATG4/CASP3 and COIIV/COX-2/NF-B Expression as a potential therapy for DAB-induced HCC. Gin was administered to HCC mice induced by p-Dimethylaminoazobenzene (DAB) alone or combined with sorafenib (Sor). Superoxide dismutase (SOD), catalase (CAT), and oxidative stress malondialdehyde (MDA), as well as biochemical markers including AST, ALT, ALP, Albumin, and Bilirubin, were examined. The expression of oncogenes (COIIV, COX-2, NF-κB, and survivin) and tumor suppressor genes (ATG4 and CASP3) was evaluated using qPCR. According to the results, the levels of MDA have been markedly decreased, while SOD and CAT have been increased. Further, the expression levels of tumor suppressor genes were upregulated, whereas the expression levels of oncogene genes were downregulated. Furthermore, in a dose-dependent manner, gingerol has shown the potential to alleviate hepatic portal vein (PV) dilatation and could offer a reliable therapy for HCC. This suggests combining the two compounds may be more effective than alone and that Gin could be a promising therapeutic option for HCC. The binding of Gin and Sor to the active sites of the target genes prevents them from functioning normally, which in turn stops the pathways from carrying out their oncogenic functions. Additionally, COX-2 inhibition reduces the production of certain pro-inflammatory compounds, which further averts oncogenesis. Conclusively, this study indicated that Gin has cytoprotective properties and anti-cancer activity that may be related to controlling oxidative stress. This effect may be achieved by suppressing the COIIV/COX-2/NF-κB pathway and upregulating the ATG4 /CASP3 pathways.

**Graphical abstract:**

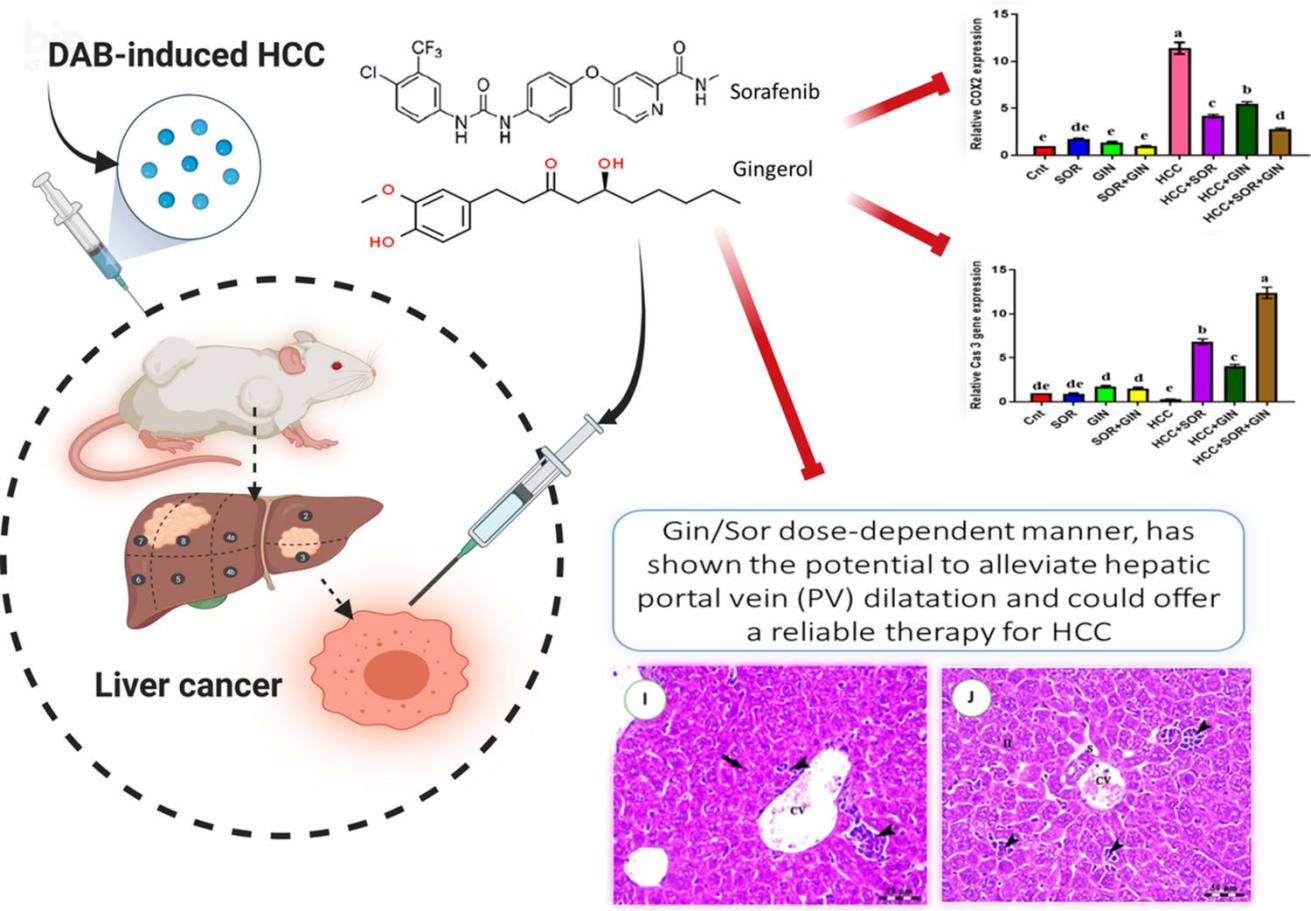

## Introduction

Hepatocellular carcinoma (HCC) accounts for around 95% of primary liver cancer cases and has emerged as a significant cause of mortality due to malignant neoplasms in Egypt. Recent data from Rashed et al. [1] indicate that HCC ranks as the third greatest cause of death among males and the fifth major cause among women in the country. The elucidation of many molecular pathways has shown their involvement in HCC development, including perturbations in the cell cycle and programmed cell death pathway [2]. Consequently, in-depth molecular investigations can potentially enhance current therapeutic approaches for cancer.

The use of animal models has been proven to be very valuable in examining the effects of p-dimethylaminoazobenzene (DAB) on hepatocarcinogenesis. Through these models, researchers have gained a deeper knowledge of how DAB might begin liver cancer at the biochemical and molecular levels [3]. It is important to note that metabolizing DAB by cytochrome P450 enzyme releases many electrophiles and reactive oxygen species (ROS). The ROS can interact with DNA, leading to conformational alterations that can potentially contribute to tumorigenesis [4]. According to a recent research conducted by Lu et al. [5], it was shown that reducing the proportion of ROS and electrophiles generated may lead to a substantial reduction in the occurrence of DNA structural alterations, often referred to as adducts.

Given the restricted range of therapeutic interventions, using dietary phytochemicals in the first phases of cancer development is the most secure approach to cancer prevention now [6, 7]. Previous studies have provided evidence to support the notion that 6-Gingerol (Gin), a primary pungent constituent found in ginger, has a diverse range of pharmacological characteristics, notably including antioxidative and anti-inflammatory attributes [8]. The latter phenomenon results from the inhibitory effect of Gin on the expression of cyclooxygenase 2 (COX-2) [9], an essential enzyme involved in synthesizing prostaglandins. COX-2 is a recognized molecular target for medications used to treat inflammation and cancer prevention [10].

In recent studies, the cytotoxic effects of Gin in cancer cell lines have been elucidated, revealing its ability to induce cell death in response to autophagy and apoptosis mediated by caspase-3 [11, 12]. Moreover, according to Liu et al. [13], Gin induces apoptosis in cancer cells and inhibits their metastatic potential. Conversely, sorafenib (Sor) is considered to be among the most extensively used pharmaceutical treatments for individuals diagnosed with HCC. The failure of Sor to effectively halt tumor growth has spurred further investigation, highlighting the urgent need for the advancement of innovative pharmacological therapies for HCC [14].

Therefore, the primary objective of this experimental investigation is to examine the therapeutic impacts of Gin and/or Sor on the expression of particular proteins linked to the development of HCC. The study focuses its attention on the ATG4/CASP3 and COIIV/COX-2/NF-kB pathways as plausible targets for mitigating liver cancer caused by exposure to DAB.

## Materials and methods

### Animals

Our study was done under the ethical approval committee of the Faculty of Science, Tanta University, Egypt, which is accepted by the institutional animal care and use committee (IACUC) and according to the animal research reporting of in vivo experiments (ARRIVE) guidelines. The study was performed on male albino mice weighing about 20–25 g and aged approximately 10 weeks. Mice were kept under controlled temperature and other environmental factors, and their diet and water were also standardized. All animal models could adapt to the animal house for one week before the experiment started.

### Experimental design

One hundred sixty mice were obtained from Theodor Bilharz Research Institute (Giza, Egypt); after the acclimatization period, mice were divided into eight groups. The control group (Cnt) contained 15 mice administered only saline. The Gin group had 15 mice administered Gin orally at a 2.5 mg/20 g dose for 30 days. The Sor group consisted of 15 mice injected with 15 mg/kg of Sor daily for 30 days. The Gin + Sor group contained 15 mice administered Gin orally and Sor through IP injection. A total of 25 mice were induced for the HCC group for 60 days by p-DAB (165 mg/kg/day) [15] and phenobarbital as a promoter (0.06 ml of 0.05%/mouse) [16]. The HCC + Sor group contained 25 mice induced for HCC and treated with Sor. The HCC + Gin group included 25 mice induced for HCC and treated with Gin. The HCC + Sor + Gin group had 25 mice induced for HCC and co-treated with Sor and Gin.

### Sampling practice

Anesthesia was administered by inhaling isoflurane (2%), and cervical dislocation was used to euthanize the mice after a 24-h fast. The blood was collected in clean tubes ready for centrifugation at 2000 g for 15 min. Serum was drawn into labeled Eppendorf and saved at -20 °C for further analysis. Immediately after animals were sacrificed, liver tissues were separated, washed, and fixed in 10% buffered formalin, while the other was homogenized for further biochemical assays or frozen for RNA extraction.

### Estimation of biochemical parameters

By using available commercial kits (Sigma-Aldrich, Cairo, Egypt), serum levels for alanine aminotransferase (ALT), aspartate aminotransferase (AST), alkaline phosphatase (ALP), albumin, and total bilirubin were measured. From previously prepared homogenate, malondialdehyde (MDA), superoxide dismutase (SOD), and catalase (CAT) were calorimetrically measured by kits obtained from (Cornell Lab, Egypt) as mentioned in the previous work [17].

### Real-time-polymerase chain reaction for the selected genes

We evaluated the expression of ATG4, CASP3, COIIV, COX-2, NF-κB, and survivin genes by qPCR. The primer sequences are given in Table 1. Total RNA was extracted using the Rneasy® Mini Kit (Qiagen, Hilden, Germany). Following RNA synthesis, 500 ng of cDNA was synthesized (i-Script cDNA synthesis kit, BioRad, Hercules, USA). A final RT-PCR reaction was performed using 25 mL of Fluocycle®II SYBR® (Euroclone, Milan, Italy), 10 ng of cDNA, and 2 mL of 10 mM forward and reverse primers. Afterward, 19 uL of nuclease-free water was added. All 35 cycles of reactions were carried out at the following temperatures: 95 °C for 5 min of initial denaturation, 95 °C for 15 min of denaturation, 55 °C for 30 min of annealing, and 72 °C for 30 min of extension [18]. The Ct values were then gathered and normalized to the housekeeping gene to determine the relative gene expression in each sample [19].

**Table 1:** Primer sequences for the selected genes

ATG4	F: 5′ CAAGGGCCATGATGTAACTTTCC 3′ R: 5′ ACCAAAGATGTATTTCTGTCAAAGCAT 3′
CASP3	F: 5′ TTCATTATTCAGGCCTGCCGAGG 3′ R: 5′ TTCATTATTCAGGCCTGCCGAGG 3′
COIIV	F: 5′ TGGTCCGAATCTGCCCTCCT 3′ R: 5′ TGTCAGCAATTAGGCAGATCAAGG 3′
COX-2	F: 5′ AGGGCCAGTCCTAGTTTTGAATA 3′ R: 5′ GTGGATGCTTCGTTAATTTGTTCAG 3′
NF-κB	F: 5′ CTCTATCAGCGGCACTGCCA 3′ R: 5′ GAAGCTGTCAGCGCGTCG 3′
Survivin	F: 5′ CCCTGTAAAGCTCTCCTGTCTGACT 3′ R: 5′ GGAGCGCACGCCCTCTTAG 3′
GAPDH (internal control)	F: 5′ TGGCAAAGTGGAGATTGTTGCC 3′ R: 5′ TGGCAAAGTGGAGATTGTTGCC 3′

### Histopathological investigation

After being taken out of the liver, the tissues were preserved in formalin for a whole day. The next step involved dehydrating each tissue with ethanol and xylene before embedding it in paraffin wax. Each section was cut using a microtome and measured approximately 5 µm. A light microscope was used throughout the examination to look for histological alterations [17].

### Molecular docking study

To ascertain Gin and Sor’s molecular interactions and scores concerning the target genes, the three-dimensional structures of the target genes were obtained from the RCSB Protein Data Bank database (https://www.rcsb.org/). Furthermore, the structures of Gin and Sor were obtained from the PubChem database, accessible at https://pubchem.ncbi.nlm.nih.gov/. In addition, the target proteins were modified by eliminating water molecules and binding ligands using the UCSF Chimera software tool. The molecular docking interaction between Gin and Sor against the target genes was conducted using the AutoDock 4.2 program and UCSF Chimera. The resulting interactions were displayed using the BIOVIA Discovery Studio software.

### Statistical analysis

Data were analyzed by GraphPad Prism 8.0 and expressed in the form of Mean ± SEM. One-way analysis of variance (ANOVA) and the Tukey test for multiple comparisons was used to analyze the data. Values with a *P* value of 0.05 or above were deemed statistically significant.

## Results

### The outcome of gingerol and/or sorafenib on liver functions

Comparatively, compared to control animals, mice with HCC given Gin and/or Sor had lower blood levels of AST, ALT, ALP, and bilirubin and significantly higher serum albumin levels. Measured blood biochemical markers showed the greatest improvement in the HCC + Gin + Sor group, followed by the HCC + Gin group and the HCC + Sor group. Also, the serum ALT levels of the Sor, Gin + Sor, and Gin groups were significantly higher than those of the control group. This suggests that the combination of Gin and Sor has a synergistic effect in reducing the levels of AST, ALT, ALP, and bilirubin and increasing serum albumin levels.

It was seen that there were no significant differences in serum AST levels between the control group and the Sor, Gin + Sor, and Sor groups. Gin, however, showed a significant increase in AST levels. In addition, there were no noticeable differences in ALP and bilirubin concentrations between the Sor, Gin + Sor, and Gin cohorts compared to the control group, as revealed in Table 2. This could be due to the fact that the Sor, Gin + Sor, and Sor groups had similar levels of AST, ALP, and bilirubin, while Gin had significantly higher levels of AST. This could be due to the higher dosage of Gin, which may have caused an increase in AST levels.

**Table 2:** Different serum parameters of the liver after treatment with gingerol and/or sorafenib

Groups	ALT (U/L)	AST (U/L)	ALP (U/L)	Albumin (U/g)	Bilirubin (U/g)
Cnt	30.21 ± 0.47^f^	34.22 ± 0.59^d^	88.50 ± 8.60^d^	4.50 ± 0.010^bc^	0.031 ± 0.010^e^
Sor	33.02 ± 1.00^e^	36.31 ± 0.59^cd^	92.30 ± 9.40^d^	4.20 ± 0.012^cd^	0.025 ± 0.010^e^
Gin	29.21 ± 0.42^e^	32.02 ± 1.02^c^	95.20 ± 6.30^d^	4.70 ± 0.011^b^	0.022 ± 0.010^e^
Sor + Gin	32.40 ± 0.89^e^	30.05 ± 1.07^de^	91.00 ± 5.20^d^	4.60 ± 0.010^b^	0.020 ± 0.010^e^
HCC	84.22 ± 0.73^a^	90.72 ± 2.73^a^	145.70 ± 13.60^a^	3.60 ± 0.010^d^	0.28 ± 0.012^a^
HCC + Sor	66.02 ± 0.56^b^	64.38 ± 0.31^b^	133.40 ± 9.80^b^	4.40 ± 0.012^c^	0.23 ± 0.010^b^
HCC + Gin	64.13 ± 1.99^c^	60.41 ± 0.89^b^	128.60 ± 6.40^b^	5.20 ± 0.009^c^	0.18 ± 0.009^c^
HCC + Sor + Gin	60.30 ± 1.39^d^	57.04 ± 2.30^c^	116.40 ± 7.20^bc^	5.30 ± 0.010^a^	0.12 ± 0.010^d^

Data were presented as Mean ± SEM. Small (a–e) letters showed a marked change at *P* ≤ 0.05. The significance was expressed by dissimilar letters in the same column

### Influence of gingerol and/or sorafenib on oxidative and antioxidative markers

When comparing the control groups to the untreated HCC mice, it is evident that the hepatic levels of the CAT and SOD are much lower in the latter, while the hepatic levels of the lipid peroxidation marker MDA are notably higher. This indicates that the HCC mice had higher levels of oxidative stress, which could lead to an increased risk of developing liver cancer. This supports the claim that antioxidants can help reduce the risk of developing liver cancer.

The group that exhibited the most favorable recovery outcomes, characterized by the lowest MDA and the highest CAT and SOD levels, was the HCC + Sor + Gin group. This was followed by the HCC + Gin group and the HCC + Sor group. Treatment with Gin and/or Sor restored these markers to levels that closely resembled those in the control groups, as in Table 3. This suggests that the Gin and/or Sor treatments restored the antioxidant enzymes to the levels necessary to protect the cells from the oxidative damage caused by free radicals.

**Table 3:** Different oxidative and nonoxidative parameters of the liver after treatment with gingerol and/or sorafenib

Groups	MDA (nM/g)	SOD (U/g)	CAT (U/g)
Cnt	40.25 ± 1.47^e^	48.67 ± 1.39^ab^	29.46 ± 0.73^b^
Sor	42.33 ± 1.42^e^	44.80 ± 1.45^b^	25.13 ± 0.68^c^
Gin	35.52 ± 1.64^e^	52.65 ± 1.57^a^	32.26 ± 0.86^a^
Sor + Gin	40.16 ± 1.50^e^	49.73 ± 1.40^ab^	29.50 ± 0.74^b^
HCC	105.29 ± 5.24^a^	11.80 ± 0.59^e^	9.76 ± 0.21^f^
HCC + Sor	87.01 ± 3.11^b^	29.04 ± 0.87^d^	15.55 ± 0.34^e^
HCC + Gin	70.34 ± 3.05^c^	35.19 ± 1.05^c^	19.18 ± 0.40^d^
HCC + Sor + Gin	57.59 ± 2.46^d^	38.42 ± 1.16^e^	23.06 ± 0.52^c^

Data were presented as Mean ± SEM. Small (a–e) letters showed a marked change at *P* ≤ 0.05. The significance was expressed by dissimilar letters in the same column

### Effect of gingerol and/or sorafenib on the expression of ATG4, CASP3, COIIV, COX-2, NF-κB, and survivin genes

**Fig. 1:**
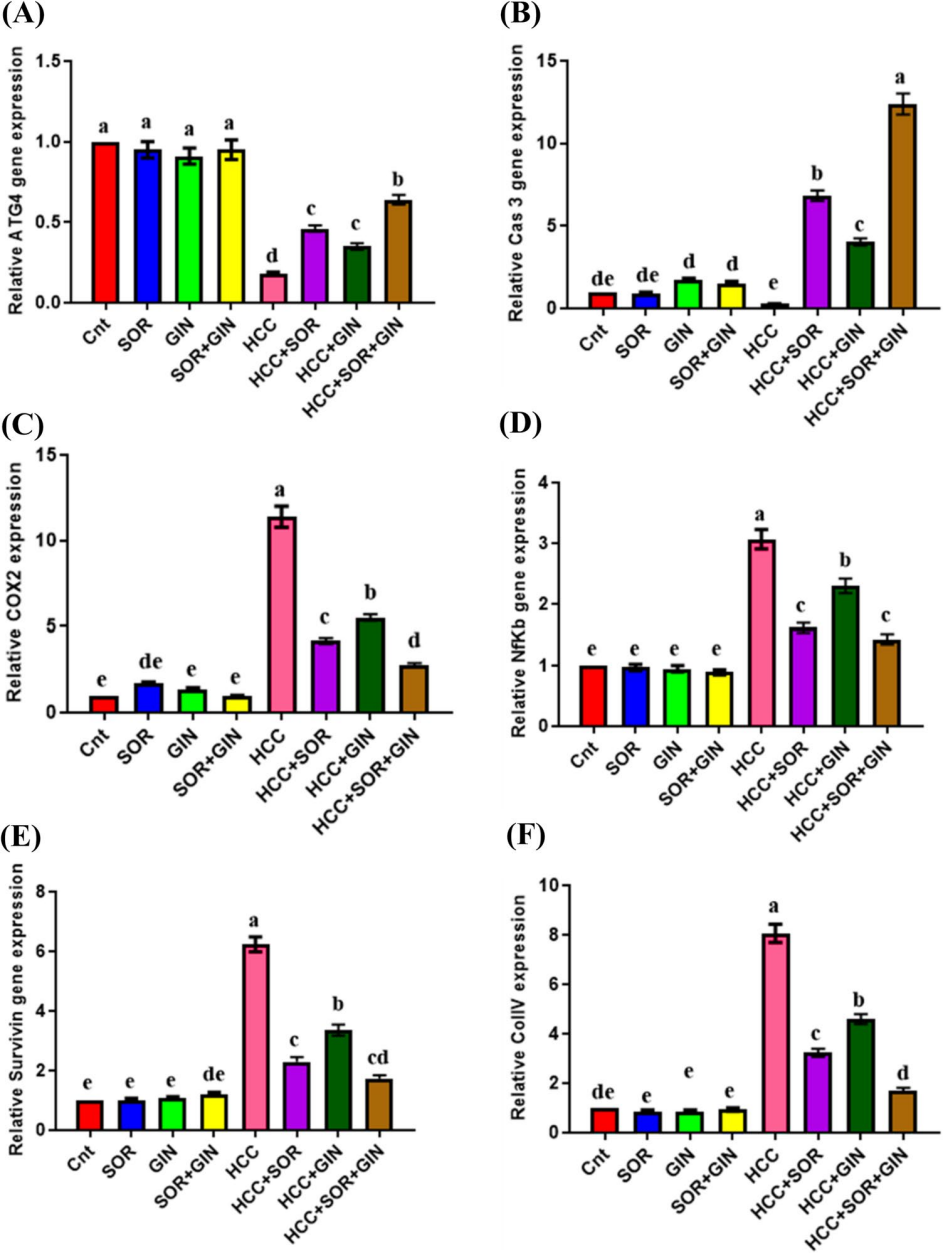
Changes in **A** ATG4, **B** CASP3, **C** COX-2, **D** NF-kB, **E** Survivin, and **F** COIIV gene expression in the liver of different groups as detected by real-time PCR. GAPDH was used as an internal control. The expression was expressed as fold changes mean ± SEM (*n* = 7/group). Columns with various letters [a (the highest fold change)–f (the lowest fold change)] showed significance at *P* < 0.05

The qPCR data indicated a substantial decrease in hepatic expression of ATG4 and CASP3 genes, as well as an increase in hepatic expression of COIIV, COX-2, NF-κB, and survivin genes in the group with HCC compared to the control groups. The administration of Gin and/or Sor enhanced gene expression, reaching levels similar to those in the control groups with the most significant improvement. Specifically, the greatest levels of ATG4 and CASP3 expression were observed in the HCC + Gin + Sor group, followed by the HCC + Sor group, and lastly, the HCC + Gin group (Fig. 1). Additionally, the expression of COIIV, COX-2, NF-κB, and survivin genes was found to be the lowest in these groups.

### Histopathological examination

**Fig. 2:**
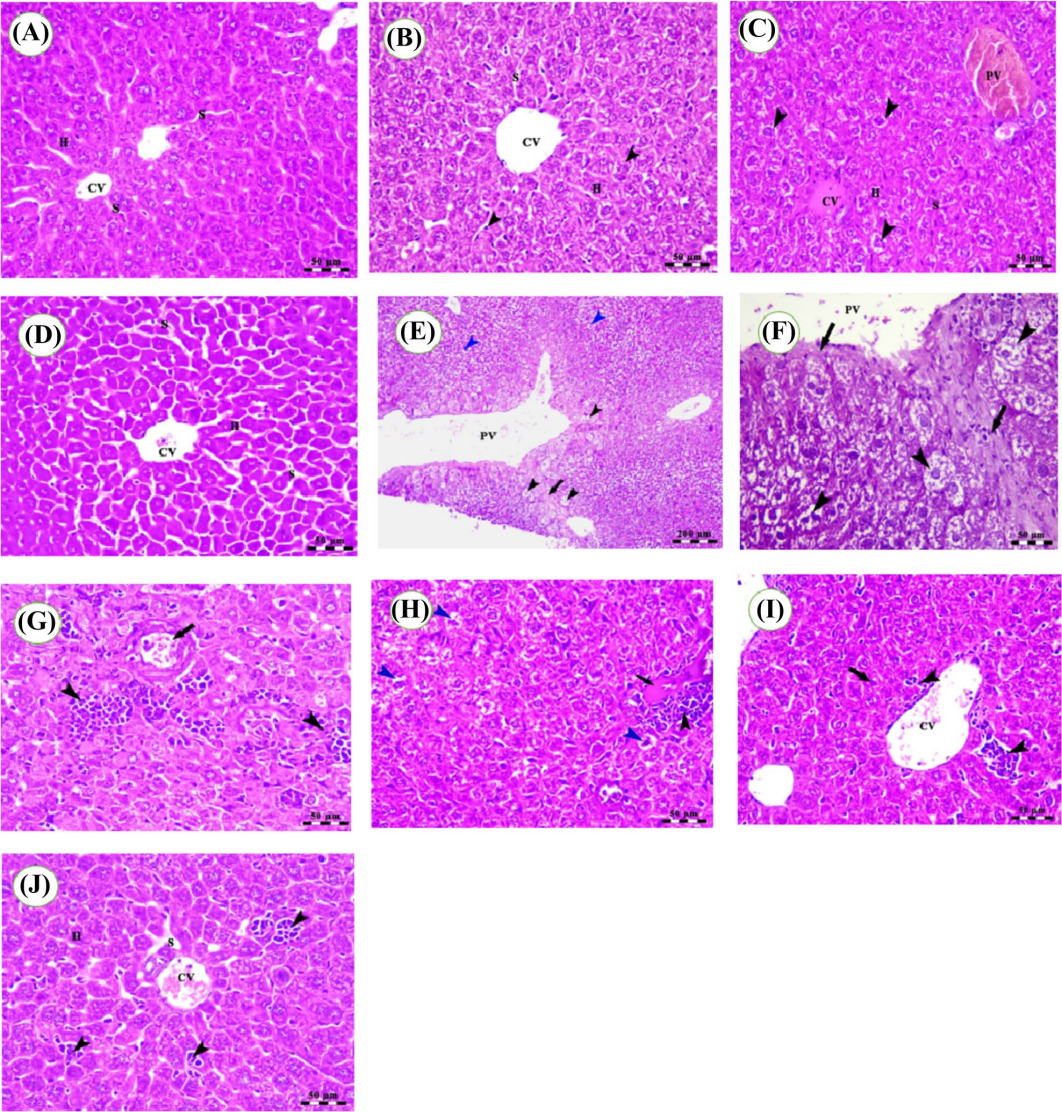
Photomicrographs of mice liver sections stained with H&E. **A** Control group shows polyhedral-shaped hepatocytes (H), blood sinusoids (S), and central vein (CV). **(B)** The Gin group shows dilated central vein (CV), polyhedral-shaped hepatocytes (H), blood sinusoids (S), and Kupffer cell activity (arrowheads). **C** Sor group shows cords of hepatocytes (H), blood sinusoids (S), dilated and congested central (CV), portal veins (PV), and vacuolar degeneration and swelling of some hepatocytes (arrowheads). **D** Gin + Sor group shows mild dilation and congestion of the central vein (CV), and blood sinusoids (S) with intact polyhedral-shaped hepatocytes (H). **E** HCC group shows dilated portal vein (PV), increased thickness of fibrous tissue (arrow), ballooning and degeneration of hepatocytes (black arrowheads), and nuclear pyknosis and vacuolar degeneration in the hepatocytes of the Centro lobular area (blue arrowheads). **F** HCC group shows dilated portal vein (PV), increased thickness of fibrous tissue (fibrosis; arrows), ballooning, and vacuolar degeneration of hepatocytes (clear cells; black arrowheads). **G** HCC group shows loss of hepatic architecture and presence of numerous aggregation of lymphoid elements (arrowheads) congestion of hepatic blood vessels (arrow). **H** HCC + Gin group shows hyalinosis in the periportal area (arrow) with the presence of perivascular lymphoid elements (black arrowheads) and vacuolar degenerative changes and ballooning of some hepatocytes (blue arrowheads). **I** HCC + Sor group shows hyalinosis in the periportal area (arrow) with the presence of perivascular lymphoid elements (black arrowheads) and vacuolar degenerative changes and ballooning of some hepatocytes (blue arrowheads). **J** HCC + Sor + Gin group shows congestion of the central vein (CV), thin hepatic trabeculae separated by mildly dilated blood sinusoids (S), and the presence of small aggregations of lymphoid elements (arrowheads). Scale bars = 50 μM. Stain H&E

A typical pattern of hepatic parenchyma can be revealed in Fig. 2A, characterized by polyhedral-shaped hepatocytes organized in a cord-like pattern. Blood sinusoids were revealed extending from the intact central vein to divide these hepatocytes. Hepatocytes in the Gin group formed a cord-like structure with blood sinusoids separating them. Additionally, there was observed activation of Kupffer cells (arrowheads) (Fig. 2B). The liver of the Sor group had hepatocytes arranged in cords, with blood sinusoids that seemed dilated and congested. Additionally, both the central and portal veins were revealed to be dilated and congested. Furthermore, vacuolar degeneration and swelling of some hepatocytes were observed (Fig. 2C). The liver of the Gin + Sor experimental group exhibited slight enlargement and increased blood flow in the central vein and blood sinusoids while maintaining the presence of hepatocytes with a polyhedral shape (Fig. 2D).

In contrast, the livers of mice induced with the HCC group exhibited various pathological features. These included the presence of dilated portal vein, increased thickness of fibrous tissue, ballooning and degeneration of hepatocytes, as well as nuclear pyknosis and vacuolar degeneration specifically observed in the hepatocytes of the Centro lobular area (Fig. 2E). Upon closer examination of Fig. 2E, it was revealed that the portal vein was dilated, the fibrous tissue exhibited increased thickness (arrows), and the hepatocytes had ballooning and vacuolar degeneration (clear cells; black arrowheads). In Fig. 2F, an additional photomicrograph of the liver from the group induced with HCC demonstrates a disruption in the liver's normal structure and many clusters of lymphoid components (arrowheads). Furthermore, there is evidence of congestion in the hepatic blood arteries (indicated by an arrow), as revealed in Fig. 2G.

The three experimental groups exhibited significant enhancements in liver morphology, characterized by reduced levels of damage compared to the HCC group. Within the HCC + Gin group, the liver exhibited hyalinosis in the periportal region, as shown by an arrow. Additionally, perivascular lymphoid components, denoted by black arrowheads, were revealed, together with vacuolar degenerative alterations and ballooning of select hepatocytes, represented by blue arrowheads (Fig. 2H). The liver of the HCC + Sor group exhibited a dilated and congested central vein, together with the thickening of hepatic trabeculae characterized by nuclear pyknosis (shown by an arrow). Additionally, tiny aggregations of lymphoid components were revealed (indicated by arrowheads) (Fig. 2I). The liver of the HCC + Sor + Gin group exhibited central vein congestion, hepatic trabeculae with a thin structure consisting of one or two rows of hepatocytes, which were separated by mildly dilated blood sinusoids. Additionally, tiny aggregations of lymphoid components were revealed (arrowheads) (Fig. 2J).

### Molecular interaction of Gin and Sor assessment

**Fig. 3:**
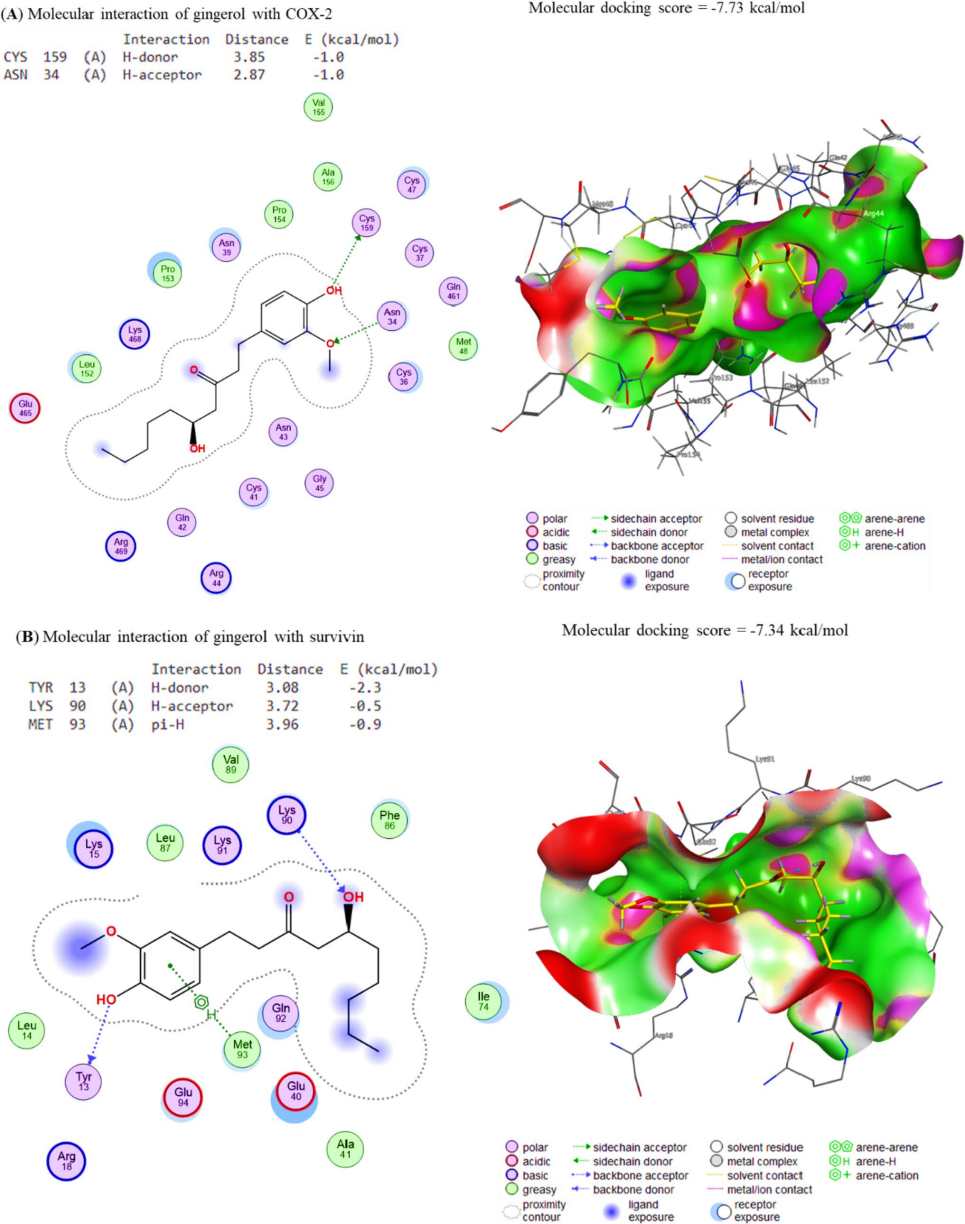
The docking interactions of Gin with **A** COX-2 and **B** Survivin. COX-2 and Survivin binding sites were bound by the compound Gin, leading to binding energies of − 7.73 and − 7.34 kcal/mol, respectively. These binding energies indicate that the docking interactions of Gin with COX-2 and Survivin were strong and stable, indicating that the compound may be able to effectively target these molecules

**Fig. 4:**
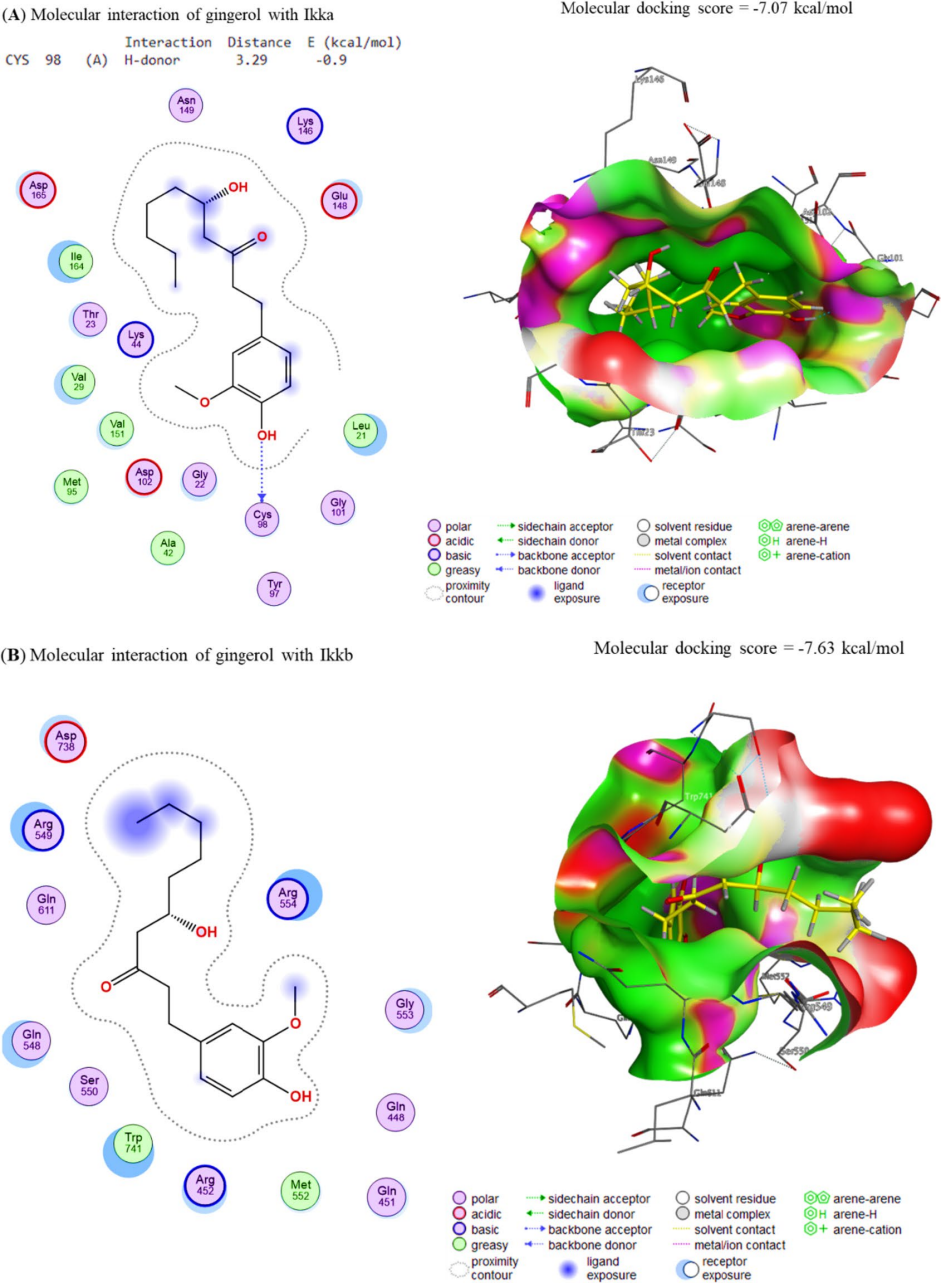

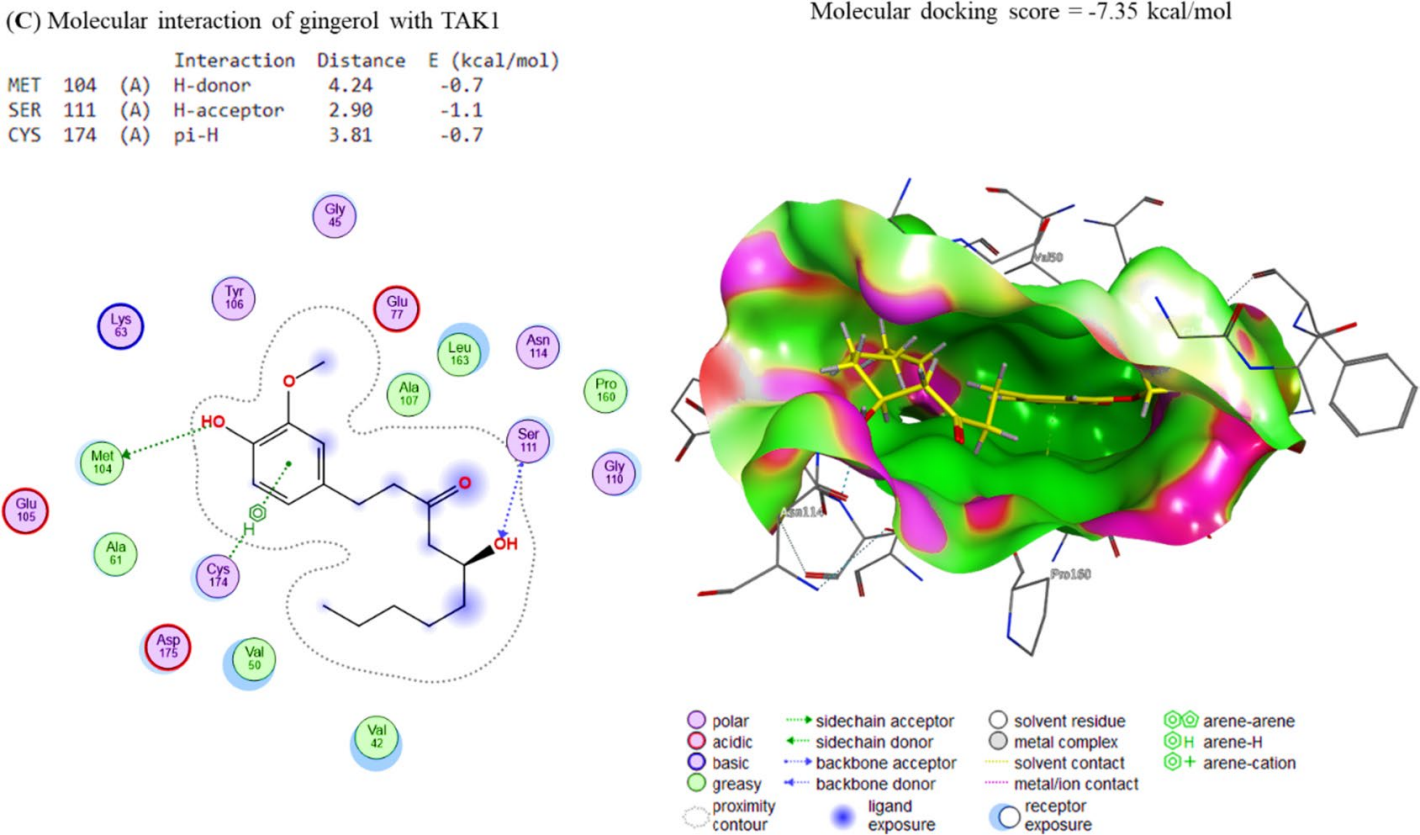
The docking interactions of Gin with **A** IKKa, **B** IKKb and **C** TAK1. The binding affinities of Gin to the IKKa, IKKb and TAK1 interaction sites were − 7.07, − 7.63, and − 7.35 kcal/mol, respectively. This indicates that Gin has a strong binding affinity for all three proteins, which suggests that it may play a role in their regulation. Additionally, the higher binding affinity of Gin to IKKa and IKKb compared to TAK1 suggests that it may act more directly on these proteins

The docking interactions between Gin and COX-2, Survivin, IKKa, IKKb, and TAK1 were elucidated correspondingly. The molecule Gin showed interactions with the binding sites of COX-2 and Survivin, leading to binding energies of − 7.73 and − 7.34 kcal/mol in Fig. 3A, B, respectively. In a similar vein, it was shown that Gin exhibited a binding affinity of − 7.07 7, 7.63, and − 7.35 kcal/mol towards the IKKa, IKKb, and TAK1 interaction sites, respectively (Fig. 4A–C). The energies that have been found indicate a notable affinity between Gin and the specific binding sites of the target genes. This implies that the chemicals found in Gin have the potential to interact with these genes, thereby hindering their respective functions.

**Fig. 5:**
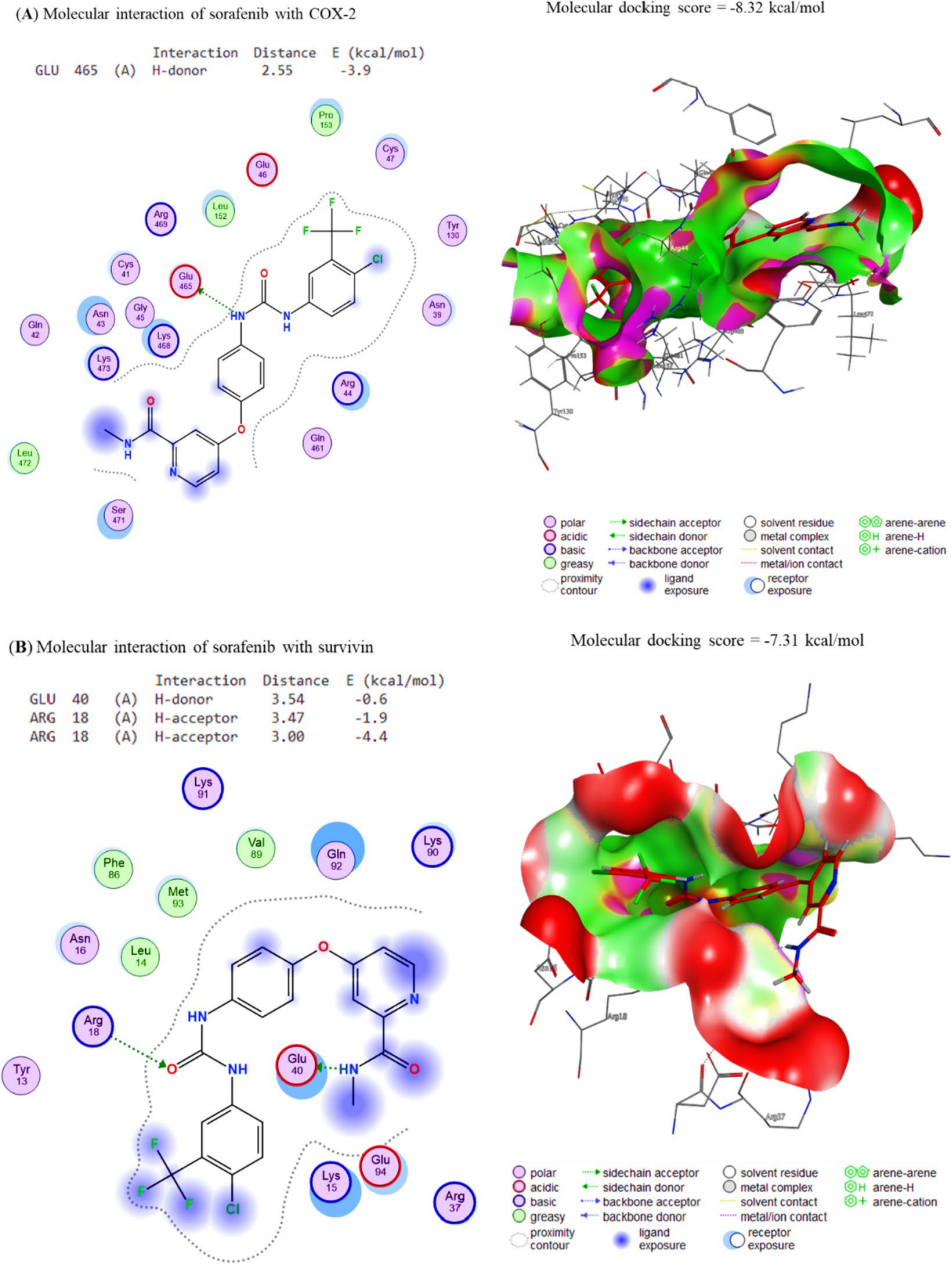
The docking interactions of Sor with **A** COX-2 and **B** Survivin. COX-2 and Survivin binding sites were bound by the compound Gin, leading to binding energies of − 8.32 and − 7.31 kcal/mol, respectively. The compound Gin binds to COX-2 and Survivin through electrostatic interactions, van der Waals interactions, and hydrogen bonding. These interactions cause the compound to bind to the proteins with enough force to stabilize the complexes and cause changes in their structure

**Fig. 6:**
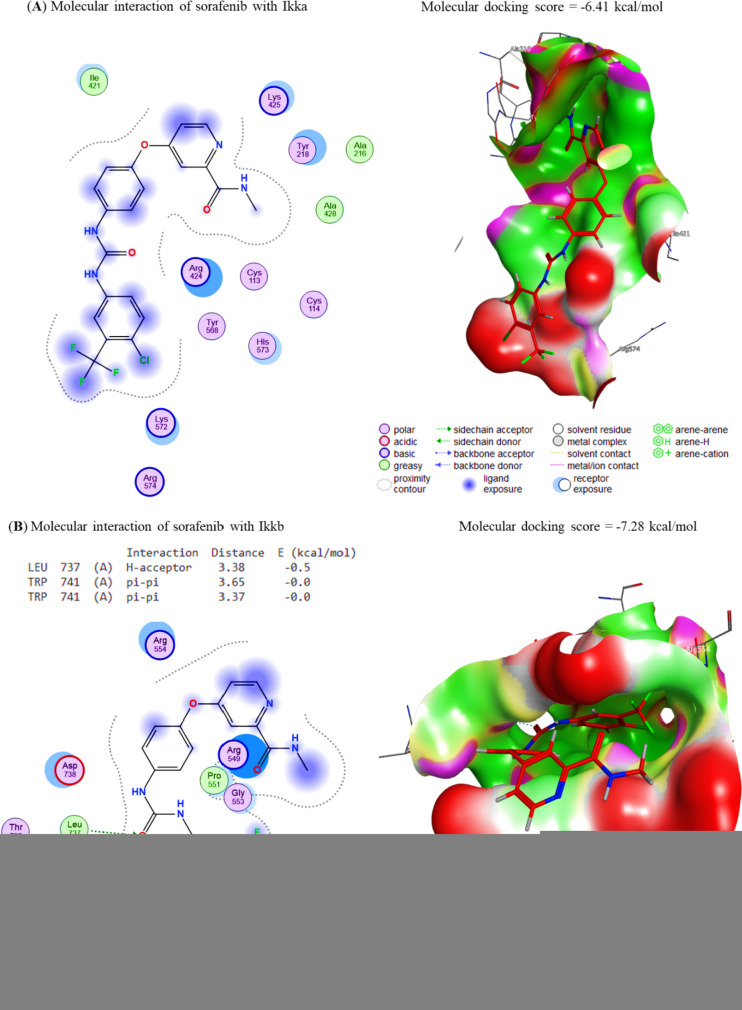

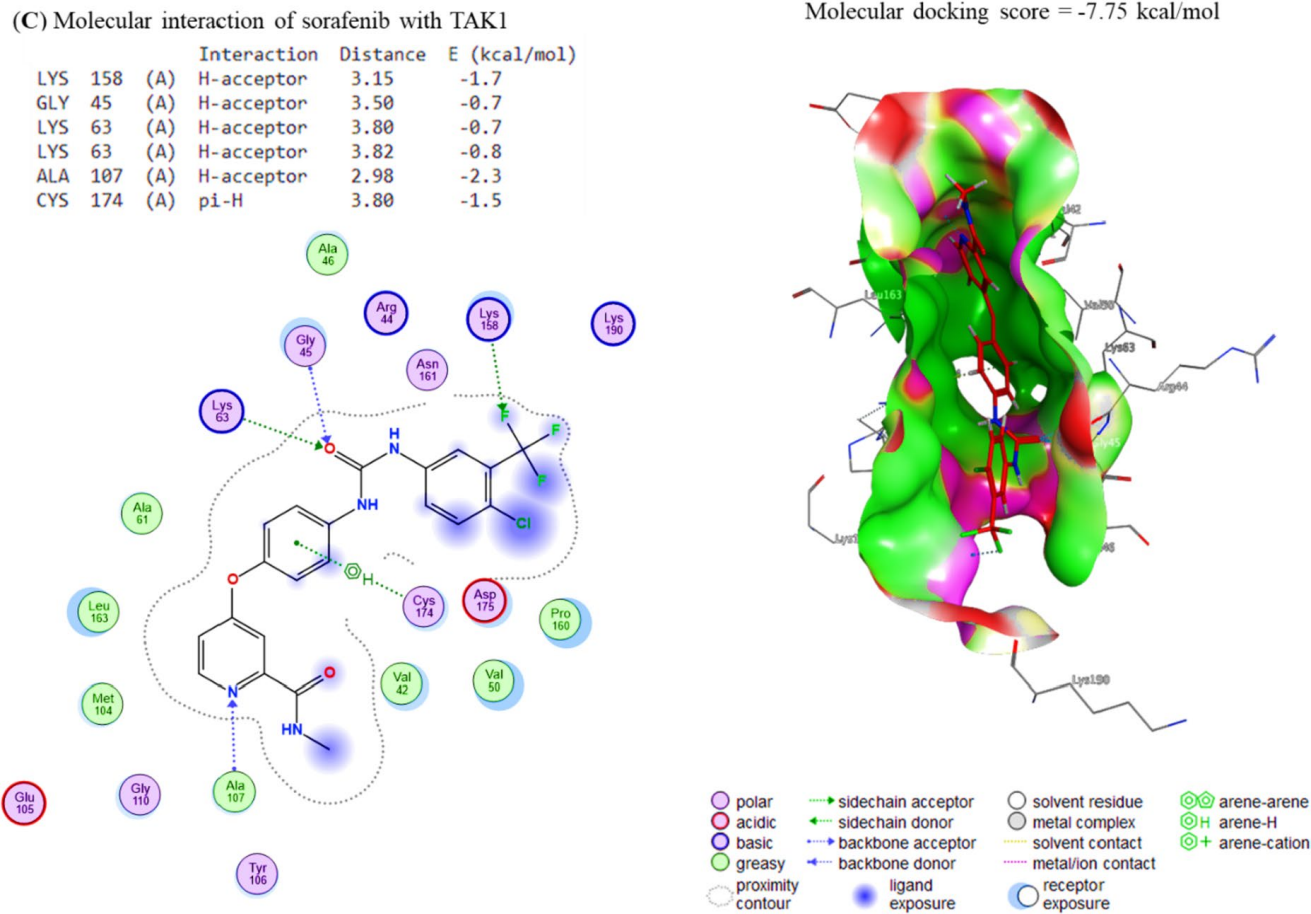
The docking interactions of Sor with **A** IKKa, **B** IKKb and **C** TAK1. The binding affinities of Sor to the IKKa, IKKb and TAK1 interaction sites were − 6.41, − 7.28, and − 7.75 kcal/mol, respectively. These values indicate that Sor binds to the IKKa, IKKb and TAK1 interaction sites with high affinity, which suggests that these proteins may be important in Sor’s mechanism of action

The docking interactions of Sor to Cox-2, Survivin, IKKa, IKKb, and TAK1 were revealed, respectively. The compound Sor exhibited interactions with Cox-2 and Survivin binding sites, with affinity energies of − 8.32 and − 7.31 kcal/mol (Fig. 5A, B), respectively, due to its interaction with these sites. In the same way, Sor binds to the IKKa, IKKb, and TAK1 binding sites, respectively, with affinities of − 6.41 kcal/mol, − 7.28 kcal/mol, and − 7.75 kcal/mol (Fig. 6A–C). Considering this, Sor could be a promising candidate for developing new drugs to treat diseases that Cox-2 and Survivin, such as inflammation and cancer cause.

## Discussion

Liver cancer is a malignancy that arises as a result of damage to hepatic cells. One potential explanation for the increased concentrations of liver enzymes in the bloodstream and bilirubin, might be attributed to this phenomenon [20]. The increased levels observed suggest the progression of carcinogenesis and show that the liver was undergoing damage accompanied by the development of preneoplastic lesions [21]. However, our research findings indicate that the mice treated with ginger/or Sor had a considerably lesser response than those treated with HCC. The observed phenomenon may be attributed to the anti-cancer action of Gin/Sor, which mitigates liver damage. Several further investigations have corroborated our results, demonstrating agreement that the concurrent administration of Sor and Gin may effectively reduce blood levels of ALT, AST, ALP, and bilirubin [22, 23].

Albumin is an essential protein that is well-recognized as a liver marker [24]. Under normal physiological conditions, the liver synthesizes this substance, but its concentration in the blood is diminished in instances of hepatic injury [25]. Our data analysis indicated a significant reduction in albumin levels within the group induced with HCC compared to the control group. However, after administration of Gin and/or Sor, there was an observed improvement in albumin levels, with the most significant increase revealed in the group treated with both Gin and Sor. These findings are consistent with the results of previous research that have been published [26–28].

Increased oxidative stress leads to cellular signaling changes contributing to cancer [29, 30]. DAB induced this mouse model of HCC to produce several markers of liver oxidative stress, including MDA. Furthermore, based on our preceding observations in HCC models [17], the livers of the HCC group in this research exhibited a distinct pattern of reduced levels of endogenous antioxidants, SOD and CAT, which are responsible for mitigating oxidative damage caused by ROS. In this study, the researchers observed that the administration of ginger to mice treated with DAB effectively mitigated the oxidative stress caused by DAB. This was assessed by examining the restoration of antioxidants to their normal levels and the reduction in MDA levels. The antioxidative stress properties of Gin are associated with a simultaneous decrease in liver damage and have revealed potential for anti-cancer action. The results, as mentioned earlier, are consistent with previous research indicating the potential preventive properties of ginger extracts in mitigating oxidative stress. This was assessed in a liver cancer model produced by ethionine [31]. Furthermore, these results are corroborated by additional research that demonstrates the efficacy of Gin in enhancing the antioxidant system and mitigating oxidative stress [32, 33].

This phenomenon might be attributed to the antioxidative characteristics shown by ginger, which can be attributed to the presence of Gin phenolic components. The antioxidant action of ginger is attributed to gingerols, a group of phenolic chemicals found in ginger. The standardized extract used in this research contained 5% gingerols [34].

At the molecular level, a significant increase in the expression of tumor suppressor genes, including ATG4 and CASP3, was revealed in the experimental groups who received treatment with Gin, Sor, or a combination of both. The co-treated group, which received both Gin and/or Sor, had the most favorable outcome. This phenomenon may be explained by the observation that these genes regulate autophagy and the modulation of the apoptotic pathway, respectively. Furthermore, our investigation revealed a considerable downregulation of oncogenes after treatment with Gin and/or Sor. The genes under consideration include COIIV, COX-2, NF-κB, and survivin genes. Several early cancer-related alterations have been demonstrated to be initiated by chronic inflammation in the form of activated inflammatory cells, primarily Kupffer cells. Chronic inflammation is accompanied by dysregulation of pro-inflammatory mediators such as COX-2 and transcription factors like nuclear transcription factor kappa B (NF-κB) [21, 35]. In the current investigation, it was observed that ginger exhibited a capacity to safeguard against liver inflammation induced by DAB. This protective effect was attributed to decreased Kupffer cells within the liver tissues. The decrease in Kupffer cell count was associated with the suppression of COX-2 protein expression, an inflammatory enzyme involved in the production of prostaglandins [36]. This suggestion indicates Gin may protect against carcinogenesis by reducing inflammation by suppressing COX-2.

Moreover, the existence of COIIV in cancerous cells is attributed to its significant involvement in promoting cellular survival and proliferation. The pro-inflammatory factor known as NF-κB is revealed to be increased in cancer since it contributes to the promotion of carcinogenesis [37]. There has been a growing body of evidence that cancer-induced inflammation in the body can be triggered as a result of elevated oxidative damage and the elevation of inflammatory markers, such as NF-κB, after carcinogenesis takes place. In addition to its role as a master regulator of inflammation, NF-κB serves as a mediator of communication between inflammation and cancer at various stages [38]. The NF-κB signaling pathway has been shown to have a significant role in promoting the proliferation of neoplastic cells, inhibiting their programmed cell death (apoptosis), and facilitating the progression and spread of malignancies [39].

It should be noted, however, that even though Gin is administered to mice treated with DAB in this study, this effectively does lower the levels of protein expressed by the NF-κB p65 subunit caused by DAB. As a result of Gin's inhibitory properties, its protective effects in ethionine-induced rat hepatoma models have been noted [40]. In the same line, Kim et al. [41] reported that gingerol, one of ginger's active components, inhibited COX-2 expression in a mouse skin cancer model by blocking the activation of NF-kB.

The histopathological analysis revealed an observed augmentation in the fibrous tissue thickness and vascular degradation of hepatocytes in the group induced with HCC. Furthermore, disruption of hepatic architecture was announced, accompanied by the accumulation of a substantial quantity of lymphoid components. Following the administration of Gin and/or Sor, a notable enhancement in the histoarchitecture of hepatic cells was observed. The study revealed a reduction in the aggregation of lower lymphoid components and a decrease in the thickness of hepatic trabeculae. This discovery was corroborated by Lamfon [42], who found that giving rats both ginger and metalaxyl enhanced the biochemical and histological alterations that metalaxyl caused in hepatocytes. Also, In a study conducted by Badr et al. [43], it was discovered that the experimental Ehrlich ascites carcinoma treated with ginger preserved normal liver architecture and histological structure, with the presence of somewhat congested and swollen blood vessels.

## Conclusions

In mice with HCC, Gin and/or Sor significantly decreased lipid peroxidation, improved antioxidant status, and increased anti-cancer activity. Considering its impact on oxidative stress and its ability to inhibit the COIIV/COX-2/NF-B pathways responsible for the initiation and progression of cancer, Gin could be an effective cancer treatment candidate. Furthermore, the results suggest Gin and/or Sor may offer a promising new strategy for treating HCC by adjuvant chemotherapy and radiation treatment.

## Data Availability

The data presented in this study are available on request from the corresponding author.

## References

[1] Rashed WM, Kandeil MAM, Mahmoud MO, Ezzat S (2020). Hepatocellular carcinoma (HCC) in Egypt: a comprehensive overview. J Egypt Natl Cancer Inst.

[2] Mohamed EE, Abdel-Moneim A, Ahmed OM (2022). Anti-cancer activity of a novel naringin-dextrin nanoformula: preparation, characterization, and in vitro induction of apoptosis in human hepatocellular carcinoma cells by inducing ROS generation, DNA fragmentation, and cell cycle arrest. J Drug Deliv Sci Technol.

[3] Biswas SJ, Bhattacharjee N, Khuda-Bukhsh AR (2008). Efficacy of a plant extract (*Chelidonium majus* L.) in combating induced hepatocarcinogenesis in mice. Food Chem Toxicol.

[4] Thomas NS, George K, Namasivayam N (2016). Molecular aspects and chemoprevention of dimethylaminoazobenzene-induced hepatocarcinogenesis: a review. Hepatol Res.

[5] Lu M, Ji J, Jiang Z, You Q (2016). The Keap1–Nrf2–ARE pathway as a potential preventive and therapeutic target: an update. Med Res Rev.

[6] Ali R, Eltantawi HG, Rizk MES (2021). Is amygdalin outcomes weighing detriments of sorafenib treatment in female mice with kidney injury induced by ehrlich ascites carcinoma model? Preliminary study. Biochem Lett.

[7] Elmalla A, Elmetwalli A, Rizk ME-S, Salama AF (2021). The effect of vitamin B17 on cardiomyopathy against Ehrlich tumor development in female mice. Biochem Lett.

[8] Mashhadi NS, Ghiasvand R, Askari G (2013). Antioxidative and anti-inflammatory effects of ginger in health and physical activity: review of current evidence. Int J Prev Med.

[9] Eren D, Betul YM (2016). Revealing the effect of 6-gingerol, 6-shogaol and curcumin on mPGES-1, GSK-3β and β-catenin pathway in A549 cell line. Chem Biol Interact.

[10] Dannenberg AJ, Altorki NK, Boyle JO (2001). Cyclooxygenase 2: a pharmacological target for the prevention of cancer. Lancet Oncol.

[11] Chakraborty D, Bishayee K, Ghosh S (2012). [6]-Gingerol induces caspase 3 dependent apoptosis and autophagy in cancer cells: drug–DNA interaction and expression of certain signal genes in HeLa cells. Eur J Pharmacol.

[12] Lalami ZA, Tafvizi F, Naseh V, Salehipour M (2022). Characterization and optimization of co-delivery Farnesol-Gingerol Niosomal formulation to enhance anti-cancer activities against breast cancer cells. J Drug Deliv Sci Technol.

[13] Liu C-M, An L, Wu Z (2022). 6-Gingerol suppresses cell viability, migration and invasion via inhibiting EMT, and inducing autophagy and ferroptosis in LPS-stimulated and LPS-unstimulated prostate cancer cells. Oncol Lett.

[14] Nasser HM, El-Naggar SA, El-Sayed Rizk ME-SR (2021). Effect of sorafenib on liver biochemistry prior to vitamin B17 coadministration in Ehrlich ascites carcinoma mice model: preliminary phase study. Biochem Lett.

[15] Karmakar SR, Biswas SJ, Khuda-Bukhsh AR (2010). Anti-carcinogenic potentials of a plant extract (*Hydrastis canadensis*): I. Evidence from in vivo studies in mice (*Mus musculus*). Asian Pac J Cancer Prev.

[16] Pathak S, Khuda-Bukhsh AR (2007). Assessment of hepatocellular damage and hematological alterations in mice chronically fed p-dimethyl aminoazobenzene and phenobarbital. Exp Mol Pathol.

[17] Attia AA, Salama AF, Eldiasty JG (2022). Amygdalin potentiates the anti-cancer effect of sorafenib on Ehrlich ascites carcinoma and ameliorates the associated liver damage. Sci Rep.

[18] Abumelha HMA, Saeed A (2020). Synthesis of some 5-arylidene-2-(4-acetamidophenylimino)-thiazolidin-4-one derivatives and exploring their breast anti-cancer activity. J Heterocycl Chem.

[19] Gad EM, Nafie MS, Eltamany EH (2020). Discovery of new apoptosis-inducing agents for breast cancer based on ethyl 2-amino-4, 5, 6, 7-tetra hydrobenzo [b] thiophene-3-carboxylate: synthesis, in vitro, and in vivo activity evaluation. Molecules.

[20] AM El-Deeb F, Mahmoud YI, Hassan NHA (2022). Re-evaluation of the anticarcinogenic effect of metformin in a chemically-induced hepatocellular carcinoma model not associated with diabetes. Egypt J Basic Appl Sci.

[21] Hamza AA, Heeba GH, Hamza S (2021). Standardized extract of ginger ameliorates liver cancer by reducing proliferation and inducing apoptosis through inhibition oxidative stress/inflammation pathway. Biomed Pharmacother.

[22] Haniadka R, Saxena A, Shivashankara AR (2013). Ginger protects the liver against the toxic effects of xenobiotic compounds: preclinical observations. J Nutr Food Sci.

[23] Helal EGE, El-Wahab A, Samia M (2012). Effect of *Zingiber officinale* on fatty liver induced by oxytetracycline in albino rats. Egypt J Hosp Med.

[24] Sitar ME, Aydin S, Cakatay U (2013). Human serum albumin and its relation with oxidative stress. Clin Lab.

[25] Moman RN, Gupta N, Varacallo M (2022). Physiology, Albumin.

[26] Abdel-Azeem AS, Hegazy AM, Ibrahim KS (2013). Hepatoprotective, antioxidant, and ameliorative effects of ginger (*Zingiber officinale* Roscoe) and vitamin E in acetaminophen treated rats. J Diet Suppl.

[27] Osama A, Fatma A, Mohamed E, Huda S (2014). Studies on the protective effects of ginger extract and in combination with ascorbic acid against aluminum toxicity induced hematological disorders, oxidative stress and hepatorenal damage in rats. Ann Vet Anim Sci.

[28] Yahyazadeh R, Baradaran Rahimi V, Yahyazadeh A (2021). Promising effects of gingerol against toxins: a review article. BioFactors.

[29] Ray PD, Huang B-W, Tsuji Y (2012). Reactive oxygen species (ROS) homeostasis and redox regulation in cellular signaling. Cell Signal.

[30] Rezatabar S, Karimian A, Rameshknia V (2019). RAS/MAPK signaling functions in oxidative stress, DNA damage response and cancer progression. J Cell Physiol.

[31] Yusof Y, Ahmad N, Das S (2009). Chemopreventive efficacy of ginger (*Zingiber officinale*) in ethionine induced rat hepatocarcinogenesis. Afr J Tradit Complement Altern Med.

[32] Cheng Y, Lu C, Yen G (2017). Phytochemicals enhance antioxidant enzyme expression to protect against NSAID-induced oxidative damage of the gastrointestinal mucosa. Mol Nutr Food Res.

[33] Dugasani S, Pichika MR, Nadarajah VD (2010). Comparative antioxidant and anti-inflammatory effects of [6]-gingerol,[8]-gingerol,[10]-gingerol and [6]-shogaol. J Ethnopharmacol.

[34] Ghasemzadeh A, Jaafar HZE, Rahmat A (2015). Optimization protocol for the extraction of 6-gingerol and 6-shogaol from *Zingiber officinale* var. *rubrum Theilade* and improving antioxidant and anti-cancer activity using response surface methodology. BMC Complement Altern Med.

[35] Lee JH. In vitro and in vivo anti-inflammatory effects of rosmanol and carnosol isolated from rosemary. Diss. Rutgers University-Graduate School-New Brunswick. 2010.

[36] Ribeiro D, Freitas M, Tomé SM (2015). Flavonoids inhibit COX-1 and COX-2 enzymes and cytokine/chemokine production in human whole blood. Inflammation.

[37] Taniguchi K, Karin M (2018). NF-κB, inflammation, immunity and cancer: coming of age. Nat Rev Immunol.

[38] Shi X, Li D, Deng Q (2015). NEFAs activate the oxidative stress-mediated NF-κB signaling pathway to induce inflammatory response in calf hepatocytes. J Steroid Biochem Mol Biol.

[39] Wang S, Liu Z, Wang L, Zhang X (2009). NF-κB signaling pathway, inflammation and colorectal cancer. Cell Mol Immunol.

[40] Habib SHM, Makpol S, Hamid NAA (2008). Ginger extract (*Zingiber officinale*) has anti-cancer and anti-inflammatory effects on ethionine-induced hepatoma rats. Clinics.

[41] Kim SO, Kundu JK, Shin YK (2005). [6]-Gingerol inhibits COX-2 expression by blocking the activation of p38 MAP kinase and NF-κB in phorbol ester-stimulated mouse skin. Oncogene.

[42] Lamfon HA (2011). Protective effect of ginger (*Zingiber officinale*) against metalaxyl induced hepatotoxicity in albino mice. J Am Sci.

[43] Badr OM, Abd-Eltawab HM, Sakr SA (2016). Ameliorative effect of ginger extract against pathological alterations induced in mice bearing solid tumors. J Biosci Appl Res.

