# Correction to: Gingerol and/or sorafenib attenuates the DAB-induced HCC and hepatic portal vein dilatation via ATG4/CASP3 and COIIV/COX-2/NF-κB expression

**DOI:** 10.1007/s12032-024-02352-2

**Published:** 2024-04-22

**Authors:** Afrah Fatthi Salama, Ali H. El-Far, Esraa Ali Anbar, Sabry Ali El-Naggar, Rami M. Elshazli, Alaa Elmetwalli

**Affiliations:** 1 Biochemistry Section, Chemistry Department, Faculty of Science, Tanta University, Tanta, 31527 Egypt; 2 Department of Biochemistry, Faculty of Veterinary Medicine, Damanhour University, Damanhour, 22511 Egypt; 3 Physiology Department, Faculty of Science, Tanta University, Tanta, 31527 Egypt; 4 Biochemistry and Molecular Genetics Unit, Department of Basic Science, Faculty of Physical Therapy, Hours University, New Damietta, Egypt; 5 Department of Clinical Trial Research Unit and Drug Discovery, Egyptian Liver Research Institute and Hospital (ELRIAH), Mansoura, Egypt; 6 Microbiology Division, Higher Technological Institute of Applied Health Sciences, Egyptian Liver Research Institute and Hospital (ELRIAH), Mansoura, Egypt

**Correction to: Medical Oncology (2024) 41:57** 10.1007/s12032-023-02284-3

The original version of this article contained an error. Following the publication of the article, the authors noticed typographical errors which should be corrected.

In the Results section (under heading ‘Influence of gingerol and/or sorafenib on oxidative and antioxidative markers’), the article originally stated incorrectly as below:

When comparing the control groups to the untreated HCC mice, it is evident that the hepatic levels of the CAT and SOD are much **higher** in the latter, while the hepatic levels of the lipid peroxidation marker MDA are notably **lower**.

The correct sentence is:

When comparing the control groups to the untreated HCC mice, it is evident that the hepatic levels of the CAT and SOD are much **lower** in the latter, while the hepatic levels of the lipid peroxidation marker MDA are notably **higher**.

In addition, Table 3 originally published with the columns MDA (nM/g) and SOD (U/g) containing identical data. Correct Table 3 has been provided below:

**Table 3:** Incorrect version

Groups	MDA (nM/g)	SOD (U/g)	CAT (U/g)
Cnt	48.67 ± 1.39^e^	48.67 ± 1.39^ab^	29.46 ± 0.73^b^
Sor	44.80 ± 1.45^e^	44.80 ± 1.45^b^	25.13 ± 0.86^c^
Gin	52.65 ± 1.57^e^	52.65 ± 1.57^a^	32.26 ± 0.86^a^
Sor + Gin	49.73 ± 1.40^e^	49.73 ± 1.40^ab^	29.50 ± 0.74^b^
HCC	11.80 ± 0.59^a^	11.80 ± 0.59^e^	9.76 ± 0.21^f^
HCC + Sor	29.04 ± 0.87^b^	29.04 ± 0.87^d^	15.55 ± 0.34^e^
HCC + Gin	35.19 ± 1.05^c^	35.19 ± 1.05^c^	19.18 ± 0.40^d^
HCC + Sor + Gin	38.42 ± 1.16^d^	38.42 ± 1.16^e^	23.06 ± 0.52^c^

Data were presented as Mean ± SEM. Small (a–e) letters showed a marked change at *P* ≤ 0.05. The significance was expressed by dissimilar letters in the same column

**Table 3:** Correct version

Groups	MDA (nM/g)	SOD (U/g)	CAT (U/g)
Cnt	40.25 ± 1.47^e^	48.67 ± 1.39^ab^	29.46 ± 0.73^b^
Sor	42.33 ± 1.42^e^	44.80 ± 1.45^b^	25.13 ± 0.86^c^
Gin	35.52 ± 1.64^e^	52.65 ± 1.57^a^	32.26 ± 0.86^a^
Sor + Gin	40.16 ± 1.50^e^	49.73 ± 1.40^ab^	29.50 ± 0.74^b^
HCC	105.29 ± 5.24^a^	11.80 ± 0.59^e^	9.76 ± 0.21^f^
HCC + Sor	87.01 ± 3.11^b^	29.04 ± 0.87^d^	15.55 ± 0.34^e^
HCC + Gin	70.34 ± 3.05^c^	35.19 ± 1.05^c^	19.18 ± 0.40^d^
HCC + Sor + Gin	57.59 ± 2.46^d^	38.42 ± 1.16^e^	23.06 ± 0.52^c^

Data were presented as Mean ± SEM. Small (a–e) letters showed a marked change at *P* ≤ 0.05. The significance was expressed by dissimilar letters in the same column

The correction does not have any effect on the results or conclusions of the paper.

The original article has been corrected.

